# Secondary syphilis concomitant with primary lesion and early neurosyphilis in a kidney transplant recipient^⋆^

**DOI:** 10.1016/j.abd.2022.12.002

**Published:** 2023-05-22

**Authors:** Ana Claudia Athanasio Shwetz, Gabriel Berg de Almeida, Ricardo de Souza Cavalcante, Juliano Vilaverde Schmitt, Luciana Patrícia Fernandes Abbade, Ricardo Augusto Monteiro de Barros Almeida

**Affiliations:** Department of Infectious Diseases, Dermatology, Diagnostic Imaging and Radiotherapy, Faculty of Medicine, Universidade Estadual Paulista, Botucatu, SP, Brazil

Dear Editor,

*Treponema pallidum,* subspecies *pallidum,* is the causative agent of syphilis. Syphilis can be transmitted through sexual contact, blood transfusion, solid organ transplantation, and from mother to child.^1^, ^2^ The rising incidence of syphilis is a global public health problem.^3^ Despite the large number of people undergoing kidney transplantations (KTx), data on syphilis in this population are scarce.^4^, ^5^, ^6^, ^7^, ^8^, ^9^

A 24-year-old man presented to the emergency room in July 2020 with a painless genital ulcer for the preceding 20 days (Fig. 1A). Subsequently, multiple non-pruritic and painless erythematous papules with peripheral desquamation appeared. These papules had a symmetrical distribution, located on the trunk (Fig. 1B), upper limbs, and palmoplantar region (Fig. 2A). He also had odynophagia and right peripheral facial palsy (Fig. 2B). He underwent kidney transplantation 18 months prior to the presentation. Immunosuppression included tacrolimus, sodium mycophenolate, and prednisone. He reported being heterosexual and denied sexual intercourse in the 3 months prior to presentation, or any previous episodes of syphilis. The serum treponemal Chemiluminescence Immunoassay (CLIA) test of the recipient before KTx was nonreactive (Table 1), as was the serum Venereal Disease Research Laboratory (VDRL) test of the deceased donor.

**Figure 1:**
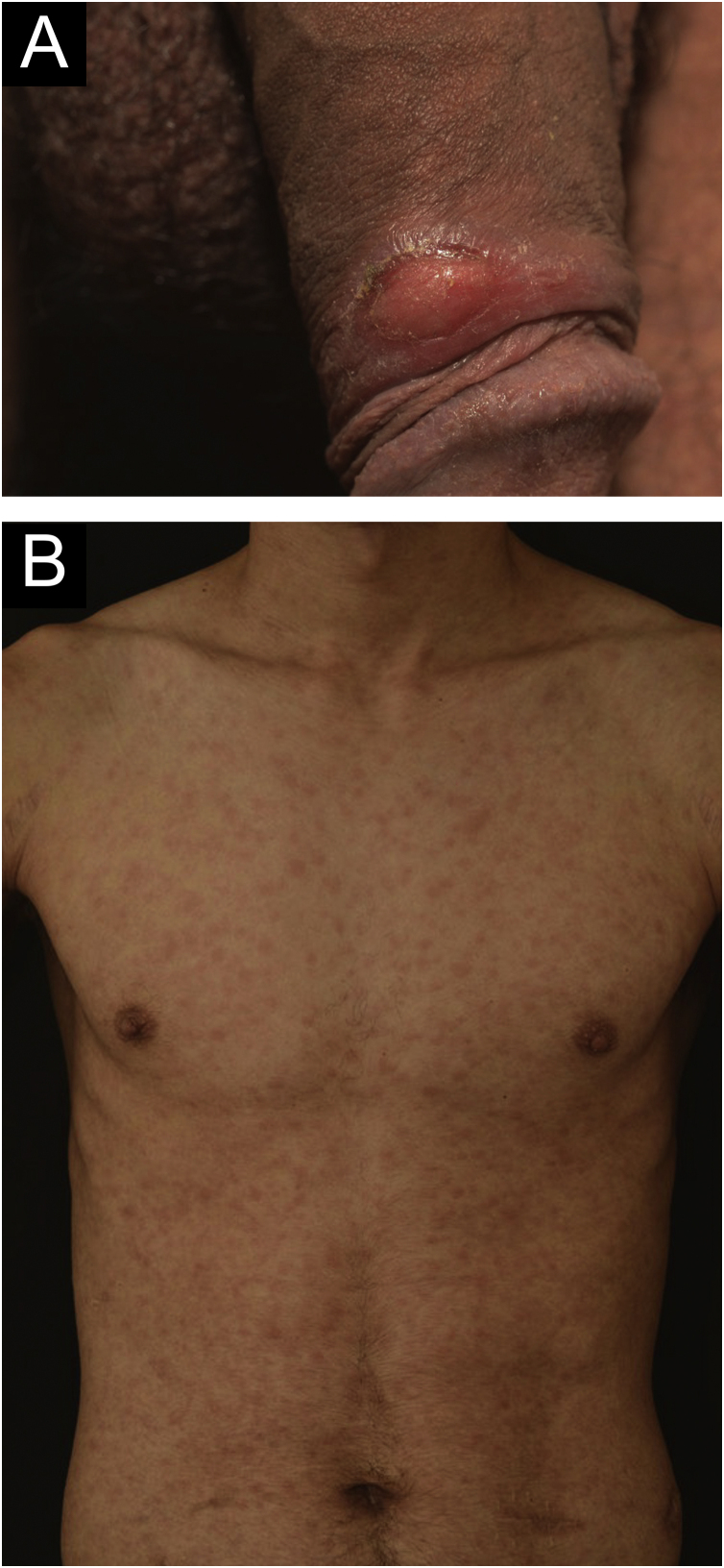
(A) Ulcer with well-defined edges, erythematous bed, located in the foreskin. (B) Multiple erythematous papules distributed on the trunk

**Figure 2:**
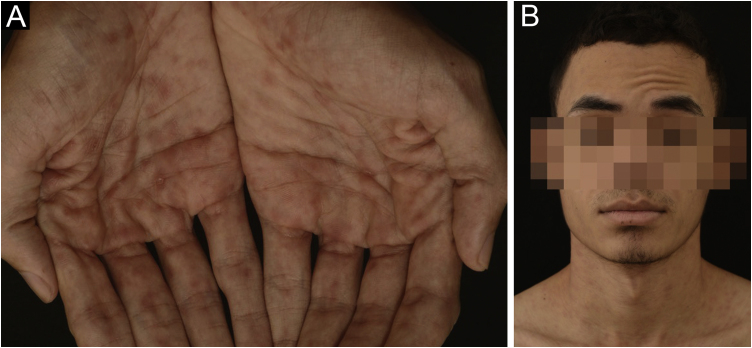
(A) Multiple erythematous papules distributed on the palms. (B) Peripheral facial nerve palsy

**Table 1:** Cerebrospinal fluid and serological data of the kidney transplant recipient

**CSF**	**2-days pre-treatment**		**13-days of treatment**		**180-days post-treatment**	
Fluid aspect	Clear and colorless		Clear and colorless		Clear and colorless	
WBC (/mm^3^)	8		5		2	
Lymphocytes (%)	60		NA		NA	
Monocytes (%)	1		NA		NA	
Neutrophils (%)	39		NA		NA	
RBC (/mm^3^)	5		15		2	
Protein (mg/dL)	75		66		37	
Glucose (mg/dL)	53		50		54	
CSF/serum glucose ratio	0.47		NA		0.62	
VDRL	Nonreactive		Nonreactive		Nonreactive	
FTA-ABS (IgM)	Nonreactive		NA		NA	
FTA-ABS (IgG)	Reactive		NA		NA	
Others (all negative)	Bacterial/fungal culture; India ink stain; cryptococcal antigen latex agglutination test; adenosine deaminase; RT-PCR: CMV, VZV, HSV-1, HSV-2		NA		NA	

**Serum**	**35 months pre-KTx**	**2 days pre-treatment**	**71 days post-treatment**	**156 days post-treatment**	**212 days post-treatment**	**338 days post-treatment**
VDRL (Titer)	NA	1:16	1:8	1:2	1:2	1:2
Treponemal CLIA	Nonreactive	Reactive	Reactive	Reactive	Reactive	Reactive

CSF, Cerebrospinal Fluid; WBC, White Blood Cell; RBC, Red Blood Cell; VDRL, Venereal Disease Research Laboratory test; FTA-ABS, Fluorescent Treponemal Antibody Absorption test; IgM, Immunoglobulin M; IgG, Immunoglobulin G; RT-PCR, Real-Time Polymerase Chain Reaction; CMV, Cytomegalovirus; VZV, Varicella-Zoster Virus; HSV, Herpes Simplex Virus; KTx, Kidney Transplantation; CLIA, Chemiluminescent Immunoassay; NA, Not Available.

At presentation, the serological investigation showed VDRL (1:16) and CLIA reactivity (Table 1). Lymphopenia (880 lymphocytes/mm^3^) and mild liver enzyme changes (Alanine aminotransferase, 59 U/L; alkaline phosphatase, 136 U/L; gamma-glutamyl transferase, 174 U/L) were identified. The estimated glomerular filtration rate decreased by 12% from baseline (93 mL/min/1.73 m^2^). Serological tests for HIV 1/2, HTLV I/II, and hepatitis B and C, as well as HIV-1 plasma viral load and RT-PCR for COVID-19, were negative. Brain computed tomography revealed no abnormalities. Cerebrospinal Fluid (CSF) analysis showed slight lymphocytic pleocytosis, elevated protein levels, and hypoglycorrhachia. CSF VDRL was non-reactive; however, the Fluorescent Treponemal Antibody Absorption Test (FTA-ABS) IgG was reactive (Table 1). The ophthalmologic evaluation revealed papilledema in the right eye. The skin biopsy from the trunk showed a pattern of interface dermatitis associated with perivascular and peri-annexal lymphocytic infiltrate, with rare plasma cells (Fig. 3A). Immunohistochemical analysis of *T. pallidum* was positive (Fig. 3B).

**Figure 3:**
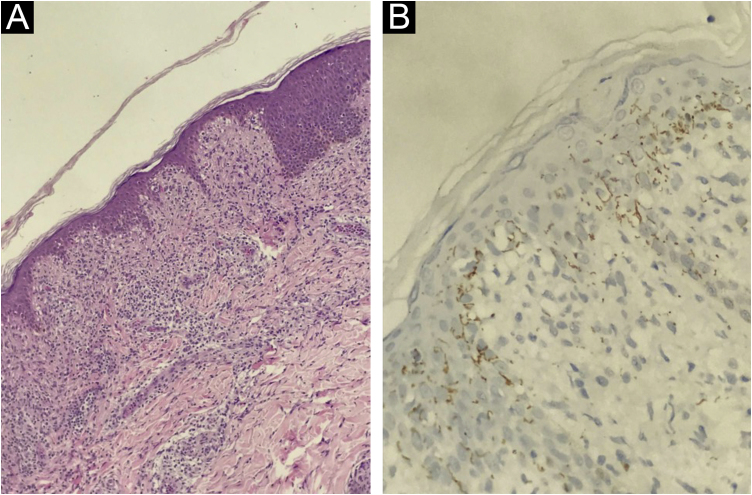
(A) Skin biopsy: histological examination by Hematoxylin & eosin, ×400, showing nonspecific lymphocytic and histiocytic infiltrate with some plasma cells. (B) Immunohistochemistry, ×400, for *Treponema pallidum* in skin biopsy showing the presence of spirochetes

The patient was diagnosed with secondary syphilis concomitant with primary lesion and early neurosyphilis and received intravenous potassium penicillin G (4 million IU every 4 h for 14 days). After the 14-day treatment ended, the penile lesion was healed, and evident improvement was noted in other dermatological lesions and facial paralysis, with normalization of liver enzyme levels and renal function. Thereafter, the immunosuppressive regimen was not modified. One month after the 14-day treatment, the patient was completely asymptomatic. Three months after the treatment, the papilledema was still in regression. At the 15-month follow-up after the 14-day treatment, there was no recurrence of syphilis, and the ophthalmologic examination no longer showed papilledema. Table 1 presents serological responses to syphilis treatment.

The diagnosis was primary-secondary syphilis (early syphilis), even with the patient's report of absence of sexual activity in the last three months, as the patient may have omitted such information, and the clinical picture was typical of lesions in the primary and secondary phases. It could be questioned whether the penile lesion was a manifestation of primary syphilis or secondary syphilis. However, the penile lesion was the first to appear, clinically compatible with primary chancre, and the lesions on the body and palm of the soles, typical of secondary syphilis, seemed only a few days later.

The natural course of sexually acquired syphilis has well-defined phases characterized sequentially by the primary, secondary, latent syphilis, and tertiary stages (up to 40%).^1^ However, the clinical presentation is often atypical in immunocompromised populations. In people living with HIV/AIDS, there is concurrence between the primary and secondary phases, rapid evolution to the tertiary stage (secondo-tertiaryism), the occurrence of multiple primary lesions, secondary malignant syphilis, early neurological, and ophthalmological involvement, involvement of cranial nerves, in addition to gastric, hepatic, and pulmonary syphilis.^10^

We identified only six reports of acquired syphilis after kidney transplantation, with severe and disseminated cases prevailing.^4^, ^5^, ^6^, ^7^, ^8^, ^9^ This case corroborated the previously described hepatic, neurological, and ophthalmic involvement.^4^, ^5^, ^6^, ^7^, ^8^, ^9^ However, it presented with the following unusual symptoms: simultaneous primary and secondary involvement, peripheral facial palsy, and false-negative VDRL in CSF.

Attention should be paid to these atypical presentations in patients undergoing KTx, as syphilis has been shown to be an even better mimic in immunocompromised patients than in immunocompetent patients.^1^

## Financial support

None declared.

## Authors’ contributions

Ana Claudia Athanasio Shwetz: Elaboração do texto, participação efetiva na propedêutica, revisão de literatura, revisão crítica e aprovação do manuscrito.

Gabriel Berg de Almeida: Elaboração do texto, participação efetiva na orientação, participação efetiva na propedêutica, revisão de literatura, revisão crítica e aprovação do manuscrito.

Ricardo de Souza Cavalcante: Elaboração do texto, participação efetiva na orientação, participação efetiva na propedêutica, revisão de literatura, revisão crítica e aprovação do manuscrito.

Juliano Vilaverde Schmitt: Elaboração do texto, participação efetiva na orientação, participação efetiva na propedêutica, revisão de literatura, revisão crítica, e aprovação do manuscrito.

Luciana Patrícia Fernandes Abbade: Elaboração do texto, participação efetiva na orientação, participação efetiva na propedêutica, revisão de literatura, revisão crítica e aprovação do manuscrito.

Ricardo Augusto Monteiro de Barros Almeida: Elaboração do texto, participação efetiva na orientação, participação efetiva na propedêutica, revisão de literatura, revisão crítica e aprovação do manuscrito.

## Conflicts of interest

None declared.
